# Supplementary material for: The Enantioselective Potential of NicoShell and TeicoShell Columns for Basic Pharmaceuticals and Forensic Drugs in Sub/Supercritical Fluid Chromatography
Source: Molecules. 2023 Jan 26;28(3):1202. doi: 10.3390/molecules28031202 (PMC9919078; doi:10.3390/molecules28031202)
Supplement: Supplementary file 1 [file molecules-28-01202-s001.zip › molecules-2162047-supplementary.pdf]

The enantioselective potential of NicoShell and TeicoShell columns for basic pharmaceuticals and forensic drugs in sub/supercritical fluid chromatography

Denisa Folprechtová[1], Martin G. Schmid[2], Daniel W. Armstrong[3], Květa Kalíková[1,*]

[1] Department of Physical and Macromolecular Chemistry, Faculty of Science, Charles University, Hlavova 8, 12843 Prague, Czech Republic

[2] Department of Pharmaceutical Chemistry, Institute of Pharmaceutical Sciences, University of Graz, Graz, Austria

[3] Department of Chemistry and Biochemistry, University of Texas at Arlington, Arlington, Texas

Table S1 Structures, abbreviations, systematic names of tested analytes

Pyrovalerone derivatives		
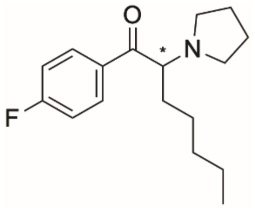	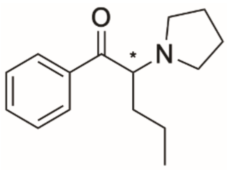	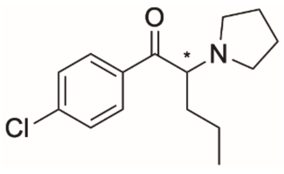
**4-F-PV8**<br>1-(4-Fluorphenyl)-2-(pyrrolidin-1-yl)heptan-1-one	***α*-PVP**<br>1-Phenyl-2-(1-pyrrolidinyl)-1-pentanone	**4-Cl-PVP**<br>1-(4-Chlorphenyl)-2-(pyrrolidin-1-yl)pentan-1-one
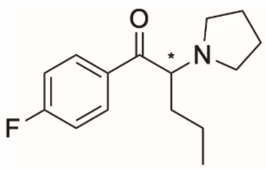	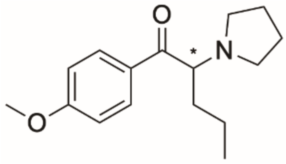	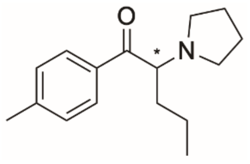
**4-F-PVP**<br>1-(4-Fluorphenyl)-2-(pyrrolidin-1-yl)pentan-1-one	**4-MeO-*α*-PVP**<br>1-(4-Methoxyphenyl)-2-(1-pyrrolidinyl)-1-pentan-1-one	**4-MPrC**<br>1-(4-Methyl-phenyl)-2-pyrrolidin-1-yl-pentan-1-one
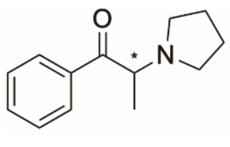	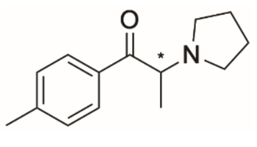	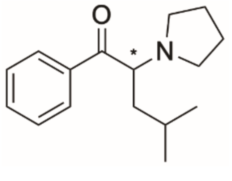
***α*-PPP**<br>1-Phenyl-2-(1-pyrrolidinyl)-1-propanone	**M-PPP**<br>1-(4-Methylphenyl)-2-(1-pyrrolidinyl)-1-propanone	***α*-PiHP**<br>4-Methyl-1-phenyl-2-(pyrrolidin-1-yl)pentan-1-one

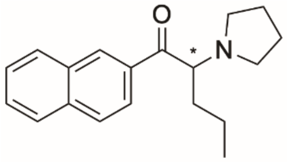	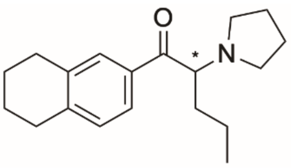	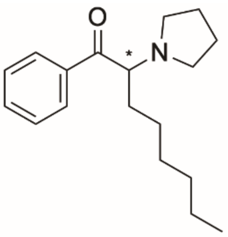
**naphyrone**<br>1-Naphthalen-2-yl-2-pyrrolidin-1-ylpentan-1-one	**TH-PVP**<br>2-(Pyrrolidin-1-yl)-1-(5,6,7,8-tetrahydronaphthalene-2-yl)pentan-1-one	**PV9**<br>1-Phenyl-2-(pyrrolidin-1-yl)octan-1-one
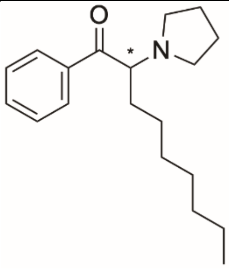	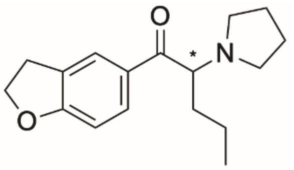	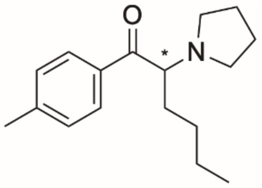
**PV10**<br>1-Phenyl-2-(pyrrolidin-1-yl)nonan-1-one	**5-DBFPV**<br>1-(2,3-Dihydrobenzofuran-5-yl)-2-(pyrrolidin-1-yl)pentan-1-on	**4-M-PHP**<br>1-(4-Methylphenyl)-2-(pyrrolidin-1-yl)hexan-1-on
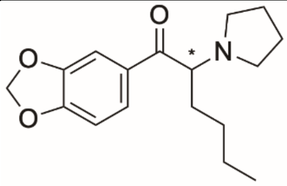		
**3,4-MD-PHP**<br>1-(1,3-Benzodioxol-5-yl)-2-(1-pyrrolidinyl)-1hexanone		
Cathinone derivatives		
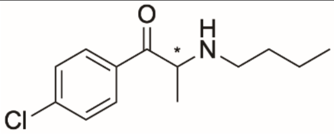	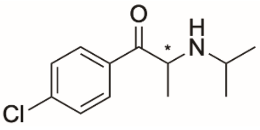	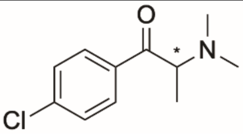
**4-CBC**<br>4-Chloro-*N*-butylcathinone	**4-CIC**<br>4-Chloro-*N*-isopropylcathinone	**4-CDC**<br>4-Chloro-*N*,*N*-dimethylcathinone
Ketamine derivatives		
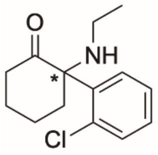	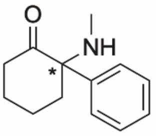	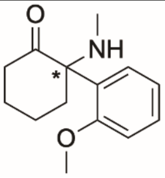
***N*-ethylketamine**<br>2-(2-Chlorophenyl)-2-(ethylamino)cyclohexan-1-on	**DXE (deschloroketamin)**<br>2-(Methylamino)-2-phenylcyclohexanone	**2-MeO-ketamine**<br>2-(2-Methoxyphenyl)-2-methylamino-cyclohexanon
Others		
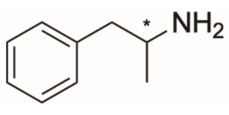	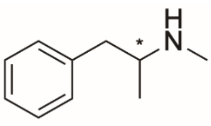	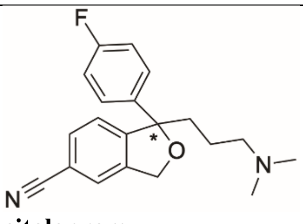
**amphetamine**	**methamphetamine**	**citalopram**

1-Phenylpropan-2-amine	*N*-Methyl-1-phenylpropan-2-amine	1-[3-(Dimethylamino)propyl]-1-(4-fluorophenyl)-1,3-dihydro-2-benzofuran-5-carbonitrile
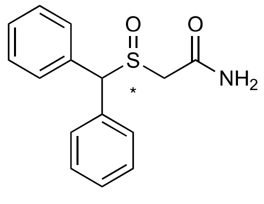	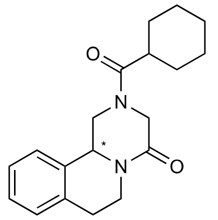	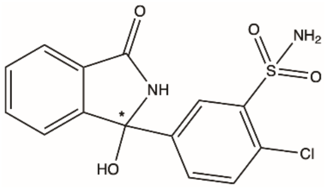
**modafinil**	**praziquantel**	**chlorthalidone**
2-[(Diphenylmethyl)sulfinyl]acetamide	2-(Cyclohexylcarbonyl)-1,2,3,6,7,11b-hexahydro-4H-pyrazino[2,1-a]isoquinolin-4-one	2-Chloro-5-(1-hydroxy-3-oxo-2,3-dihydro-1H-isoindol-1-yl)benzenesulfonamide

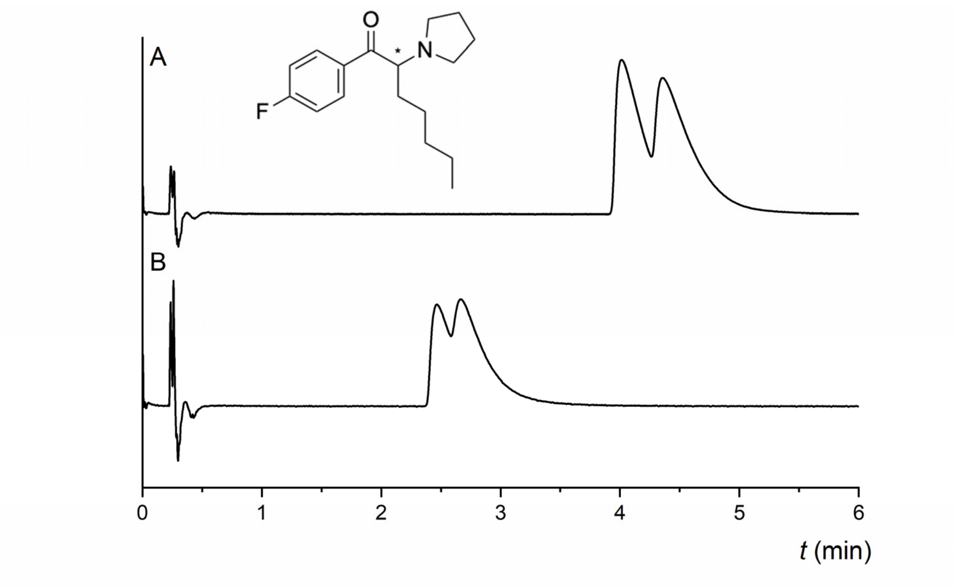

Figure S1. The effect of organic modifier on the enantioseparation of pyrovalerone 4-F-PV8 on the NicoShell column. MP composition: CO_2/MeOH (80/20, *v*/*v*) (A) and CO_2/EtOH (80/20, *v*/*v*) (B). SFC conditions: flow rate 1.00 mL min^{-1}; column temperature 25°C; BP 13.8 MPa; UV detection 254 nm.

Table S2. The data obtained from measurements on the NicoShell column under various MP compositions. Retention time of the first eluted (t_{R1}), resolution (R), enantioselectivity (α); BP 13.79 MPa; UV detection 254 or 280 nm; injection volume 0.2-1.0 µL. NOTE: C – carbon dioxide, M – methanol, E – ethanol, P – propane-2-ol, A – acetonitrile, S – slight indication of enantioseparation, O – not eluted within 30 min.

	**MP composition**	**Flow rate (mL min^{-1})**	**Temperature (°C)**
**MP →**	**C/M/AcA/TEA 90/10/0.5/0.01**	**1.00**	**40**
**Analyte ↓**	**t_{R1} (min)**	***R***	**α**
4-F-PV8	2.02	0.00	1.00
α-PVP	4.09	0.00	1.00
4-Cl-PVP	2.57	0.00	1.00
4-F-PVP	3.05	S	1.03
4-MeO-α-PVP	7.97	0.00	1.00
4-MPrC	6.97	0.67	1.05
α-PPP	9.03	3.32	1.26
M-PPP	9.72	2.91	1.21

α-PiHP	1.47	0.98	1.10
naphyrone	6.97	0.80	1.06
TH-PVP	6.98	0.67	1.05
PV9	2.99	S	1.04
PV10	2.94	S	1.03
5-DBFPV	11.38	0.50	1.06
4-M-PHP	4.21	0.00	1.00
3,4-MD-PHP	1.39	0.65	1.08
4-CBC	3.87	0.00	1.00
4-CIC	2.00	S	1.04
4-CDC	1.37	0.63	1.08
N-ethylketamine	1.25	1.50	1.18
DXE	8.39	0.00	1.00
2-MeO-ketamine	4.81	0.00	1.00
amphetamine	O		
methamphetamine	O		
citalopram	18.24	0.63	1.05
modafinil	4.03	3.80	1.31
praziquantel	0.86	0.95	1.12
chlorthalidone	O		
	**MP composition**	**Flow rate (mL min^{-1})**	**Temperature (°C)**
**MP →**	**C/M/AcA/TEA 90/10/0.2/0.02**	**1.00**	**40**
**Analyte ↓**	***t*$_{R1}$ (min)**	***R***	***α***
4-F-PV8	1.03	0.00	1.00
α-PVP	1.89	0.00	1.00
4-Cl-PVP	1.58	0.00	1.00
4-F-PVP	1.27	0.00	1.00
4-MeO-*α*-PVP	4.13	0.00	1.00
4-MPrC	3.65	0.67	1.11
α-PPP	4.57	3.45	1.30
M-PPP	5.19	3.00	1.24
α-PiHP	0.82	0.67	1.09
naphyrone	3.55	0.73	1.06
TH-PVP	3.65	0.67	1.11
PV9	1.59	0.00	1.00
PV10	1.56	0.00	1.00
5-DBFPV	6.05	0.53	1.10
4-M-PHP	2.37	0.00	1.00
3,4-MD-PHP	0.75	0.55	1.09
4-CBC	2.41	0.00	1.00
4-CIC	1.07	0.00	1.00
4-CDC	0.76	0.55	1.10
N-ethylketamine	0.76	0.93	1.17
DXE	4.58	0.00	1.00
2-MeO-ketamine	2.60	0.00	1.00

amphetamine	O		
methamphetamine	O		
citalopram	13.31	0.62	1.05
modafinil	4.03	S	1.37
praziquantel	0.81	0.83	1.10
chlorthalidone	O		
	**MP composition**	**Flow rate (mL min⁻¹)**	**Temperature (°C)**
**MP →**	**C/M/TFA/DEA 90/10/0.05/0.05**	**1.00**	**40**
**Analyte ↓**	**t_{R1} (min)**	***R***	**α**
4-F-PV8	2.66	S	1.01
α-PVP	3.78	S	1.02
4-Cl-PVP	3.11	S	1.03
4-F-PVP	3.79	0.00	1.00
4-MeO-*α*-PVP	3.00	S	1.03
4-MPrC	4.72	0.00	1.00
α-PPP	2.87	0.00	1.00
M-PPP	5.04	0.00	1.00
α-PiHP	1.87	0.82	1.04
naphyrone	5.79	0.96	1.05
TH-PVP	4.59	S	1.03
PV9	2.99	S	1.03
PV10	2.92	S	1.02
5-DBFPV	6.00	S	1.03
4-M-PHP	3.16	0.00	1.00
3,4-MD-PHP	3.20	0.00	1.00
4-CBC	1.40	0.00	1.00
4-CIC	3.54	0.00	1.00
4-CDC	3.19	0.00	1.00
N-ethylketamine	2.02	S	1.03
DXE	3.59	0.00	1.00
2-MeO-ketamine	2.69	0.00	1.00
amphetamine	3.15	0.00	1.00
methamphetamine	2.08	0.00	1.00
citalopram	4.89	0.98	1.05
modafinil	4.17	3.04	1.20
praziquantel	0.90	0.78	1.10
chlorthalidone	O		
	**MP composition**	**Flow rate (mL min⁻¹)**	**Temperature (°C)**
**MP →**	**C/M/DEA 90/10/0.1**	**1.00**	**40**
**Analyte ↓**	**t_{R1} (min)**	***R***	**α**
4-F-PV8	0.34	0.00	1.00
α-PVP	0.46	0.00	1.00
4-Cl-PVP	0.38	0.00	1.00
4-F-PVP	0.43	0.00	1.00

4-MeO-α-PVP	0.79	0.00	1.00
4-MPrC	0.75	0.00	1.00
α-PPP	0.86	1.51	1.18
M-PPP	0.95	1.26	1.17
α-PiHP	0.33	0.00	1.00
naphyrone	0.76	0.00	1.00
TH-PVP	0.75	0.00	1.00
PV9	0.43	0.00	1.00
PV10	0.42	0.00	1.00
5-DBFPV	1.15	S	1.06
4-M-PHP	0.49	0.00	1.00
3,4-MD-PHP	0.32	0.00	1.00
4-CBC	0.58	0.00	1.00
4-CIC	0.37	0.00	1.00
4-CDC	0.32	0.00	1.00
N-ethylketamine	0.39	0.00	1.00
DXE	1.07	0.00	1.00
2-MeO-ketamine	0.55	0.00	1.00
amphetamine	5.34	0.00	1.00
methamphetamine	3.18	0.00	1.00
citalopram	2.62	0.95	1.10
modafinil	3.62	6.60	1.06
praziquantel	0.64	0.68	1.13
chlorthalidone	O		

	**MP composition**	**Flow rate (mL min⁻¹)**	**Temperature (°C)**
**MP →**	**C/M/TEA 90/10/0.1**	**1.00**	**40**
**Analyte ↓**	**t_{R1} (min)**	***R***	***α***
4-F-PV8	0.42	0.00	1.00
α-PVP	0.64	0.00	1.00
4-Cl-PVP	0.49	0.00	1.00
4-F-PVP	0.55	0.00	1.00
4-MeO-α-PVP	1.19	0.00	1.00
4-MPrC	1.12	0.00	1.00
α-PPP	1.28	3.30	1.34
M-PPP	1.44	3.00	1.29
α-PiHP	0.39	0.00	1.00
naphyrone	1.10	S	1.03
TH-PVP	1.12	0.00	1.00
PV9	0.55	0.00	1.00
PV10	0.55	0.00	1.00
5-DBFPV	1.65	S	1.03
4-M-PHP	0.70	0.00	1.00
3,4-MD-PHP	0.38	0.00	1.00
4-CBC	0.96	0.00	1.00
4-CIC	0.46	0.00	1.00

4-CDC	0.39	0.00	1.00
N-ethylketamine	0.45	0.00	1.00
DXE	1.38	0.00	1.00
2-MeO-ketamine	0.86	0.00	1.00
amphetamine	9.39	0.00	1.00
methamphetamine	7.87		
citalopram	5.14	0.71	1.06
modafinil	5.14	5.13	1.50
praziquantel	0.70	S	1.13
chlorthalidone	O		
	**MP composition**	**Flow rate (mL min^{-1})**	**Temperature (°C)**
**MP →**	**C/M/IPA 90/10/0.1**	**1.00**	**40**
**Analyte ↓**	**t_{R1} (min)**	***R***	**α**
4-F-PV8	0.31	0.00	1.00
α-PVP	0.41	0.00	1.00
4-Cl-PVP	0.35	0.00	1.00
4-F-PVP	0.37	0.00	1.00
4-MeO-α-PVP	0.61	0.00	1.00
4-MPrC	0.59	0.00	1.00
α-PPP	0.68	1.02	1.17
M-PPP	0.74	0.83	1.15
α-PiHP	0.31	0.00	1.00
naphyrone	0.61	0.00	1.00
TH-PVP	0.59	0.00	1.00
PV9	0.37	0.00	1.00
PV10	0.37	0.00	1.00
5-DBFPV	0.89	0.00	1.00
4-M-PHP	0.42	0.00	1.00
3,4-MD-PHP	0.31	0.00	1.00
4-CBC	0.50	0.00	1.00
4-CIC	0.34	0.00	1.00
4-CDC	0.31	0.00	1.00
N-ethylketamine	0.37	0.00	1.00
DXE	0.92	0.00	1.00
2-MeO-ketamine	0.47	0.00	1.00
amphetamine	3.82	0.00	1.00
methamphetamine	2.50	0.00	1.00
citalopram	2.07	0.89	1.05
modafinil	3.43	5.78	1.48
praziquantel	0.62	S	1.10
chlorthalidone	O		
	**MP composition**	**Flow rate (mL min^{-1})**	**Temperature (°C)**
**MP →**	**C/M/TFA/DEA 90/10/0.2/0.02**	**1.00**	**40**
**Analyte ↓**	**t_{R1} (min)**	***R***	**α**

4-F-PV8	2.68	S	1.03
α-PVP	3.85	S	1.02
4-Cl-PVP	3.80	0.00	1.00
4-F-PVP	3.08	S	1.04
4-MeO-*α*-PVP	4.80	0.00	1.00
4-MPrC	4.72	S	1.02
α-PPP	5.58	S	1.02
M-PPP	5.08	0.00	1.00
α-PiHP	1.88	0.66	1.04
naphyrone	5.97	0.89	1.05
TH-PVP	4.73	S	1.02
PV9	3.06	S	1.03
PV10	2.98	S	1.02
5-DBFPV	6.19	S	1.01
4-M-PHP	3.23	0.00	1.00
3,4-MD-PHP	3.27	0.00	1.00
4-CBC	1.32	0.00	1.00
4-CIC	3.61	0.00	1.00
4-CDC	3.27	0.00	1.00
N-ethylketamine	1.98	S	1.03
DXE	3.50	0.00	1.00
2-MeO-ketamine	2.66	0.00	1.00
amphetamine	3.02	0.00	1.00
methamphetamine	2.00	0.00	1.00
citalopram	4.72	1.02	1.05
modafinil	4.07	2.77	1.18
praziquantel	1.00	0.80	1.10
chlorthalidone	O		

	**MP composition**	**Flow rate (mL min^{-1})**	**Temperature (°C)**
**MP →**	**C/M/A/TFA/DEA 90/8/2/0.1/0.01**	**1.00**	**40**
**Analyte ↓**	t_{R1} **(min)**	***R***	***α***
4-F-PV8	3.00	S	1.01
α-PVP	4.33	0.00	1.00
4-Cl-PVP	4.31	0.00	1.00
4-F-PVP	3.45	S	1.03
4-MeO-*α*-PVP	5.49	0.00	1.00
4-MPrC	5.50	0.00	1.00
α-PPP	6.37	0.00	1.00
M-PPP	5.81	0.00	1.00
α-PiHP	2.06	S	1.02
naphyrone	6.75	0.68	1.05
TH-PVP	5.42	S	1.01
PV9	5.45	0.00	1.00
PV10	3.35	S	1.02
5-DBFPV	7.17	0.00	1.00

4-M-PHP	3.62	0.00	1.00
3,4-MD-PHP	3.55	0.00	1.00
4-CBC	1.54	0.00	1.00
4-CIC	3.93	0.00	1.00
4-CDC	3.54	0.00	1.00
N-ethylketamine	2.18	S	1.01
DXE	3.99	0.00	1.00
2-MeO-ketamine	2.93	0.00	1.00
amphetamine	3.93	0.00	1.00
methamphetamine	2.35	0.00	1.00
citalopram	4.80	0.99	1.05
modafinil	4.85	2.83	1.20
praziquantel	1.11	0.94	1.11
chlorthalidone	O		
	**MP composition**	**Flow rate (mL min[-1])**	**Temperature (°C)**
**MP →**	**C/M/AcA/IPA 90/10/0.1/0.01**	**2.00**	**40**
**Analyte ↓**	**t_{R1} (min)**	***R***	**α**
4-F-PV8	0.32	0.00	1.00
α-PVP	0.55	0.00	1.00
4-Cl-PVP	0.38	0.00	1.00
4-F-PVP	0.47	0.00	1.00
4-MeO-α-PVP	1.15	0.00	1.00
4-MPrC	1.05	S	1.03
α-PPP	1.32	2.42	1.34
M-PPP	1.50	1.85	1.29
α-PiHP	0.28	S	1.07
naphyrone	0.98	S	1.06
TH-PVP	1.05	S	1.02
PV9	0.57	0.00	1.00
PV10	0.46	0.00	1.00
5-DBFPV	1.72	0.00	1.00
4-M-PHP	0.61	0.00	1.00
3,4-MD-PHP	0.26	0.00	1.00
4-CBC	0.62	0.00	1.00
4-CIC	0.34	0.00	1.00
4-CDC	0.26	0.00	1.00
N-ethylketamine	0.28	0.00	1.00
DXE	1.35	0.00	1.00
2-MeO-ketamine	0.68	0.00	1.00
amphetamine	6.84	0.00	1.00
methamphetamine	4.65	0.00	1.00
citalopram	3.92	0.70	1.07
modafinil	1.70	4.20	1.50
praziquantel	0.33	0.75	1.15
chlorthalidone	24.78	S	1.10

	MP composition	Flow rate (mL min-1)	Temperature (°C)
**MP →**	**C/M 90/10**	**2.00**	**40**
**Analyte ↓**	**t_{R1} (min)**	***R***	***α***
4-F-PV8	2.01	S	1.03
α-PVP	4.12	0.00	1.00
4-Cl-PVP	2.42	S	1.08
4-F-PVP	3.30	0.00	1.00
4-MeO-*α*-PVP	9.02	0.00	1.00
4-MPrC	8.00	S	1.04
α-PPP	10.89	1.94	1.38
M-PPP	12.37	1.71	1.33
α-PiHP	1.28	0.55	1.10
naphyrone	6.96	S	1.05
TH-PVP	7.68	S	1.04
PV9	2.95	0.00	1.00
PV10	2.68	0.00	1.00
5-DBFPV	13.95	0.00	1.00
4-M-PHP	3.85	0.00	1.00
3,4-MD-PHP	1.09	S	1.08
4-CBC	4.44	S	1.09
4-CIC	1.66	0.51	1.08
4-CDC	1.09	S	1.08
N-ethylketamine	0.84	0.51	1.09
DXE	7.79	S	1.06
2-MeO-ketamine	3.65	0.00	1.00
amphetamine	O		
methamphetamine	O		
citalopram	10.04	0.00	1.00
modafinil	1.88	2.73	1.30
praziquantel	0.39	0.71	1.13
chlorthalidone	23.28	0.00	1.00

	MP composition	Flow rate (mL min-1)	Temperature (°C)
**MP →**	**C/M/TFA/TEA 90/10/0.05/0.05**	**1.00**	**40**
**Analyte ↓**	**t_{R1} (min)**	***R***	***α***
4-F-PV8	2.42	0.00	1.00
α-PVP	3.51	0.00	1.00
4-Cl-PVP	2.76	S	1.02
4-F-PVP	2.75	S	1.02
4-MeO-*α*-PVP	4.36	0.00	1.00
4-MPrC	4.29	S	1.01
α-PPP	5.22	0.00	1.00
M-PPP	4.65	0.00	1.00
α-PiHP	3.12	S	1.03
naphyrone	5.44	0.69	1.04

TH-PVP	4.28	S	1.01
PV9	2.75	S	1.02
PV10	2.72	S	1.01
5-DBFPV	5.71	0.00	1.00
4-M-PHP	2.91	0.00	1.00
3,4-MD-PHP	2.98	0.00	1.00
4-CBC	1.36	0.00	1.00
4-CIC	3.26	0.00	1.00
4-CDC	2.97	0.00	1.00
N-ethylketamine	1.89	0.00	1.00
DXE	3.39	0.00	1.00
2-MeO-ketamine	2.54	0.00	1.00
amphetamine	3.16	0.00	1.00
methamphetamine	2.02	0.00	1.00
citalopram	4.45	1.01	1.05
modafinil	3.94	3.05	1.20
praziquantel	0.94	0.78	1.09
chlorthalidone	O		
	**MP composition**	**Flow rate (mL min^{-1})**	**Temperature (°C)**
**MP →**	**C/M 80/20**	**1.00**	**25**
**Analyte ↓**	***t*$_{R1}$ (min)**	***R***	***α***
4-F-PV8	4.02	0.74	1.09
α-PVP	7.46	0.00	1.00
4-Cl-PVP	5.09	0.93	1.14
4-F-PVP	5.61	0.00	1.00
4-MeO-*α*-PVP	13.77	S	1.03
4-MPrC	11.76	S	1.03
α-PPP	16.46	3.07	1.59
M-PPP	16.45	2.67	1.50
α-PiHP	6.28	0.00	1.00
naphyrone	11.84	S	1.05
TH-PVP	11.24	S	1.04
PV9	5.60	0.00	1.00
PV10	5.29	0.00	1.00
5-DBFPV	18.95	0.00	1.00
4-M-PHP	6.94	0.00	1.00
3,4-MD-PHP	2.54	0.85	1.14
4-CBC	7.58	S	1.06
4-CIC	3.31	0.50	1.07
4-CDC	2.56	0.86	1.14
N-ethylketamine	1.67	S	1.06
DXE	10.91	0.61	1.09
2-MeO-ketamine	6.01	0.00	1.00
amphetamine	O		
methamphetamine	22.41	0.00	1.00

citalopram	21.95	S	1.07
modafinil	1.52	2.58	1.36
praziquantel	0.46	S	1.15
chlorthalidone	7.67	0.00	1.00
	**MP composition**	**Flow rate (mL min^{-1})**	**Temperature (°C)**
**MP →**	**C/M/H_2O 80/20/0.5**	**1.00**	**25**
**Analyte ↓**	**t_{R1} (min)**	***R***	***α***
4-F-PV8	3.73	0.85	1.07
α-PVP	7.19	0.00	1.00
4-Cl-PVP	4.93	1.15	1.12
4-F-PVP	5.18	0.00	1.00
4-MeO-*α*-PVP	12.67	S	1.01
4-MPrC	10.64	S	1.03
α-PPP	16.28	4.64	1.51
M-PPP	15.72	4.20	1.42
α-PiHP	6.21	0.00	1.00
naphyrone	11.63	0.50	1.05
TH-PVP	10.74	S	1.03
PV9	5.17	0.00	1.00
PV10	5.06	0.00	1.00
5-DBFPV	18.07	0.00	1.00
4-M-PHP	6.85	0.00	1.00
3,4-MD-PHP	2.82	1.00	1.12
4-CBC	7.34	1.06	1.09
4-CIC	3.64	0.56	1.06
4-CDC	2.81	0.99	1.12
N-ethylketamine	1.77	S	1.05
DXE	11.69	0.99	1.09
2-MeO-ketamine	6.28	0.00	1.00
amphetamine	27.37	0.00	1.00
methamphetamine	20.14	0.00	1.00
citalopram	20.07	S	1.07
modafinil	1.26	2.65	1.29
praziquantel	0.44	S	1.17
chlorthalidone	5.65	0.00	1.00
	**MP composition**	**Flow rate (mL min^{-1})**	**Temperature (°C)**
**MP →**	**C/M/H_2O 80/20/1.0**	**1.00**	**25**
**Analyte ↓**	**t_{R1} (min)**	***R***	***α***
4-F-PV8	3.34	0.91	1.10
α-PVP	7.36	0.00	1.00
4-Cl-PVP	5.04	1.55	1.09
4-F-PVP	5.02	0.00	1.00
4-MeO-*α*-PVP	12.06	0.00	1.00
4-MPrC	10.03	0.00	1.00

α-PPP	15.68	5.74	1.44
M-PPP	14.86	5.25	1.36
α-PiHP	6.20	0.00	1.00
naphyrone	11.03	0.57	1.04
TH-PVP	9.97	0.00	1.00
PV9	5.01	0.00	1.00
PV10	4.73	0.00	1.00
5-DBFPV	16.19	0.00	1.00
4-M-PHP	6.51	0.00	1.00
3,4-MD-PHP	3.01	1.25	1.10
4-CBC	6.18	1.74	1.11
4-CIC	3.70	0.76	1.06
4-CDC	2.86	1.20	1.10
N-ethylketamine	1.80	S	1.04
DXE	11.30	1.30	1.08
2-MeO-ketamine	6.26	0.00	1.00
amphetamine	23.14	0.00	1.00
methamphetamine	17.39	0.00	1.00
citalopram	17.60	0.65	1.06
modafinil	1.12	2.56	1.25
praziquantel	0.43	0.00	1.00
chlorthalidone	4.60	0.00	1.00
	**MP composition**	**Flow rate (mL min^{-1})**	**Temperature (°C)**
**MP →**	**C/M/H_2O 80/20/1.25**	**1.00**	**25**
**Analyte ↓**	***t_{R1}* (min)**	***R***	***α***
4-F-PV8	3.42	0.68	1.05
α-PVP	7.13	0.00	1.00
4-Cl-PVP	4.76	1.50	1.10
4-F-PVP	4.69	0.00	1.00
4-MeO-α-PVP	11.10	0.00	1.00
4-MPrC	9.08	0.00	1.00
α-PPP	14.77	5.92	1.39
M-PPP	13.45	4.15	1.33
α-PiHP	5.96	0.00	1.00
naphyrone	10.06	0.58	1.04
TH-PVP	8.95	0.00	1.00
PV9	4.73	0.00	1.00
PV10	4.36	0.00	1.00
5-DBFPV	14.58	0.00	1.00
4-M-PHP	6.13	0.00	1.00
3,4-MD-PHP	2.94	1.23	1.09
4-CBC	5.74	1.84	1.11
4-CIC	3.79	0.78	1.05
4-CDC	2.95	1.24	1.09
N-ethylketamine	1.76	S	1.03

DXE	10.67	1.36	1.08
2-MeO-ketamine	6.05	0.00	1.00
amphetamine	20.39	0.00	1.00
methamphetamine	15.63	0.00	1.00
citalopram	15.35	0.71	1.06
modafinil	1.06	2.40	1.22
praziquantel	0.31	0.00	1.00
chlorthalidone	4.07	0.00	1.00
	**MP composition**	**Flow rate (mL min⁻¹)**	**Temperature (°C)**
**MP →**	**C/M/H_2O 80/20/1.5**	**1.00**	**25**
**Analyte ↓**	t_{R1} **(min)**	***R***	***α***
4-F-PV8	3.33	S	1.03
α-PVP	7.00	0.00	1.00
4-Cl-PVP	4.66	1.26	1.07
4-F-PVP	4.49	0.00	1.00
4-MeO-*α*-PVP	9.96	0.00	1.00
4-MPrC	8.15	0.00	1.00
α-PPP	13.49	4.07	1.36
M-PPP	11.94	2.96	1.31
α-PiHP	5.77	0.00	1.00
naphyrone	9.40	S	1.04
TH-PVP	8.02	0.00	1.00
PV9	4.51	0.00	1.00
PV10	4.11	0.00	1.00
5-DBFPV	12.99	0.00	1.00
4-M-PHP	5.81	0.00	1.00
3,4-MD-PHP	3.10	0.00	1.00
4-CBC	5.36	1.86	1.12
4-CIC	3.99	0.71	1.05
4-CDC	3.08	1.16	1.08
N-ethylketamine	1.86	S	1.02
DXE	10.31	1.02	1.08
2-MeO-ketamine	5.96	0.00	1.00
amphetamine	17.94	0.00	1.00
methamphetamine	14.22	0.00	1.00
citalopram	13.64	0.72	1.05
modafinil	1.02	2.11	1.19
praziquantel	0.41	0.00	1.00
chlorthalidone	3.74	0.00	1.00
	**MP composition**	**Flow rate (mL min⁻¹)**	**Temperature (°C)**
**MP →**	**C/M/TFA/IPA 90/10/0.1/0.01**	**1.00**	**25**
**Analyte ↓**	t_{R1} **(min)**	***R***	***α***
4-F-PV8	2.47	S	1.03
α-PVP	3.56	S	1.10

4-Cl-PVP	3.69	0.00	1.00
4-F-PVP	2.84	0.62	1.12
4-MeO-α-PVP	4.50	0.00	1.00
4-MPrC	4.31	S	1.04
α-PPP	5.23	S	1.03
M-PPP	4.60	0.00	1.00
α-PiHP	3.27	0.62	1.04
naphyrone	5.67	0.86	1.07
TH-PVP	4.46	S	1.04
PV9	2.87	0.64	1.10
PV10	2.80	0.53	1.10
5-DBFPV	5.96	0.00	1.00
4-M-PHP	3.00	S	1.10
3,4-MD-PHP	3.15	S	1.03
4-CBC	1.22	0.00	1.00
4-CIC	3.38	0.00	1.00
4-CDC	3.15	S	1.03
N-ethylketamine	1.73	S	1.03
DXE	3.10	S	1.03
2-MeO-ketamine	2.40	0.00	1.00
amphetamine	2.88	0.00	1.00
methamphetamine	1.88	0.00	1.00
citalopram	3.89	0.95	1.07
modafinil	3.99	2.58	1.23
praziquantel	0.89	0.64	1.12
chlorthalidone	O		
	**MP composition**	**Flow rate (mL min^{-1})**	**Temperature (°C)**
**MP →**	**C/M/H_2O/TFA/IPA 90/10/0.5/0.1/0.01**	**1.00**	**25**
**Analyte ↓**	***t_{R1}* (min)**	***R***	***α***
4-F-PV8	2.21	0.00	1.00
α-PVP	3.72	0.00	1.00
4-Cl-PVP	3.42	0.00	1.00
4-F-PVP	2.64	0.00	1.00
4-MeO-α-PVP	4.22	S	1.10
4-MPrC	3.92	0.00	1.00
α-PPP	5.35	0.00	1.00
M-PPP	4.55	0.00	1.00
α-PiHP	3.27	0.00	1.00
naphyrone	5.13	0.55	1.03
TH-PVP	3.94	0.00	1.00
PV9	2.65	0.00	1.00
PV10	2.51	0.00	1.00
5-DBFPV	5.37	0.00	1.00
4-M-PHP	2.87	0.00	1.00
3,4-MD-PHP	3.21	0.00	1.00

4-CBC	1.23	0.00	1.00
4-CIC	3.57	0.00	1.00
4-CDC	3.21	0.00	1.00
N-ethylketamine	2.04	S	1.03
DXE	3.41	0.00	1.00
2-MeO-ketamine	2.75	0.00	1.00
amphetamine	2.70	0.00	1.00
methamphetamine	1.92	0.00	1.00
citalopram	3.49	0.65	1.03
modafinil	3.01	2.68	1.14
praziquantel	0.78	0.00	1.00
chlorthalidone	22.40	0.00	1.00
	**MP composition**	**Flow rate (mL min^{-1})**	**Temperature (°C)**
**MP →**	**C/M/AcA/TEA 90/10/0.1/0.01**	**1.00**	**25**
**Analyte ↓**	**t_{R1} (min)**	***R***	***α***
4-F-PV8	1.92	0.00	1.00
α-PVP	3.81	0.00	1.00
4-Cl-PVP	2.37	0.73	1.11
4-F-PVP	2.88	0.00	1.00
4-MeO-*α*-PVP	7.73	0.00	1.00
4-MPrC	6.69	0.39	1.04
α-PPP	8.22	4.31	1.61
M-PPP	8.91	3.86	1.49
α-PiHP	3.33	0.00	1.00
naphyrone	6.91	S	1.05
TH-PVP	6.76	S	1.04
PV9	2.87	0.82	1.18
PV10	2.86	0.86	1.17
5-DBFPV	11.30	S	1.04
4-M-PHP	3.99	0.00	1.00
3,4-MD-PHP	1.27	1.02	1.17
4-CBC	4.12	0.00	1.00
4-CIC	1.17	S	1.05
4-CDC	1.27	1.03	1.17
N-ethylketamine	1.12	1.16	1.18
DXE	7.40	S	1.05
2-MeO-ketamine	4.14	0.00	1.00
amphetamine	O		
methamphetamine	O		
citalopram	16.39	0.55	1.06
modafinil	3.81	S	1.46
praziquantel	0.71	0.72	1.16
chlorthalidone	O		
	**MP composition**	**Flow rate (mL min^{-1})**	**Temperature (°C)**

MP →	C/M/H_2O/AcA/TEA 90/10/0.5/0.1/0.01	1.00	25
**Analyte ↓**	**t_{R1} (min)**	***R***	***α***
4-F-PV8	1.63	0.00	1.00
α-PVP	3.62	0.00	1.00
4-Cl-PVP	2.23	0.87	1.07
4-F-PVP	2.47	0.00	1.00
4-MeO-*α*-PVP	7.58	0.00	1.00
4-MPrC	6.09	S	1.03
α-PPP	9.61	0.75	1.47
M-PPP	9.93	6.60	1.38
α-PiHP	3.12	0.00	1.00
naphyrone	6.35	0.78	1.04
TH-PVP	6.22	S	1.03
PV9	2.46	0.00	1.00
PV10	2.28	0.00	1.00
5-DBFPV	11.30	0.00	1.00
4-M-PHP	3.52	0.00	1.00
3,4-MD-PHP	1.37	1.50	1.12
4-CBC	3.01	0.00	1.00
4-CIC	1.83	0.70	1.06
4-CDC	1.33	1.53	1.12
N-ethylketamine	1.08	0.70	1.07
DXE	7.98	1.62	1.12
2-MeO-ketamine	4.25	0.00	1.00
amphetamine	26.27	0.00	1.00
methamphetamine	17.31	0.00	1.00
citalopram	15.06	1.16	1.05
modafinil	1.34	1.55	1.12
praziquantel	0.64	0.00	1.00
chlorthalidone	O		

	MP composition	Flow rate (mL min^{-1})	Temperature (°C)
**MP →**	**C/M/H_2O 80/20/1.0**	**1.00**	**35**
**Analyte ↓**	**t_{R1} (min)**	***R***	***α***
4-F-PV8	2.81	0.78	1.05
α-PVP	5.91	0.00	1.00
4-Cl-PVP	3.45	1.27	1.09
4-F-PVP	4.05	0.00	1.00
4-MeO-*α*-PVP	10.84	0.00	1.00
4-MPrC	8.63	S	1.03
α-PPP	14.03	6.06	1.38
M-PPP	12.56	4.85	1.32
α-PiHP	5.01	0.00	1.00
naphyrone	9.18	0.59	1.04
TH-PVP	8.82	0.00	1.00
PV9	4.07	0.00	1.00

PV10	3.82	0.00	1.00
5-DBFPV	15.07	0.00	1.00
4-M-PHP	5.53	0.00	1.00
3,4-MD-PHP	2.24	1.14	1.09
4-CBC	5.21	1.10	1.09
4-CIC	3.02	0.81	1.10
4-CDC	2.24	1.15	1.09
N-ethylketamine	1.44	0.00	1.00
DXE	9.34	1.16	1.08
2-MeO-ketamine	5.36	S	1.10
amphetamine	27.09	0.00	1.00
methamphetamine	21.13	0.00	1.00
citalopram	19.87	0.80	1.05
modafinil	1.09	2.42	1.22
praziquantel	0.43	0.00	1.00
chlorthalidone	4.40	0.00	1.00
	**MP composition**	**Flow rate (mL min^{-1})**	**Temperature (°C)**
**MP →**	**C/M/TFA/DEA 90/10/0.1/0.05**	**1.00**	**40**
**Analyte ↓**	**t_{R1} (min)**	***R***	***α***
4-F-PV8	2.54	S	1.01
α-PVP	3.64	S	1.02
4-Cl-PVP	3.66	0.00	1.00
4-F-PVP	2.91	S	1.03
4-MeO-*α*-PVP	4.53	0.00	1.00
4-MPrC	4.41	S	1.02
α-PPP	5.23	0.00	1.00
M-PPP	4.74	0.00	1.00
α-PiHP	1.78	0.66	1.06
naphyrone	5.54	0.92	1.13
TH-PVP	4.40	S	1.03
PV9	2.09	S	1.03
PV10	2.81	S	1.03
5-DBFPV	5.74	S	1.02
4-M-PHP	3.03	0.00	1.00
3,4-MD-PHP	2.01	0.00	1.00
4-CBC	1.27	0.00	1.00
4-CIC	3.32	0.00	1.00
4-CDC	3.01	0.00	1.00
N-ethylketamine	1.89	S	1.03
DXE	3.26	0.00	1.00
2-MeO-ketamine	2.49	0.00	1.00
amphetamine	2.03	0.00	1.00
methamphetamine	1.88	0.00	1.00
citalopram	4.47	1.00	1.05
modafinil	4.09	2.92	1.18

praziquantel	0.98	0.71	1.10
chlorthalidone	O		

Table S3. The data obtained from measurements on the TeicoShell column under various MP compositions. Retention time of the first eluted (t_{R1}), resolution (R), enantioselectivity (α); BP 13.79 MPa; UV detection 254 or 280 nm; injection volume 0.2-1.0 µL. NOTE: C – carbon dioxide, M – methanol, E – ethanol, P – propane-2-ol, A – acetonitrile, S – slight indication of enantioseparation, X – not measured, O – not eluted within 30 min.

	**MP composition**	**Flow rate (mL min^{-1})**	**Temperature (°C)**
**MP →**	**C/M/AcA/IPA 90/10/0.1/0.01**	**2.00**	**40**
**Analyte ↓**	**t_{R1} (min)**	***R***	***α***
4-F-PV8	0.97	0.00	1.00
α-PVP	1.45	0.00	1.00
4-Cl-PVP	0.86	0.00	1.00
4-F-PVP	1.19	0.00	1.00
4-MeO-*α*-PVP	3.75	0.00	1.00
4-MPrC	3.21	0.00	1.00
α-PPP	3.90	S	1.02
M-PPP	4.64	S	1.02
α-PiHP	1.31	0.00	1.00
naphyrone	2.56	0.00	1.00
TH-PVP	3.23	0.00	1.00
PV9	1.21	0.00	1.00
PV10	1.19	0.00	1.00
5-DBFPV	5.48	0.00	1.00
4-M-PHP	1.69	0.00	1.00
3,4-MD-PHP	0.55	0.00	1.00
4-CBC	1.15	0.00	1.00
4-CIC	0.84	0.00	1.00
4-CDC	0.55	0.00	1.00
N-ethylketamine	0.42	0.00	1.00
DXE	2.50	0.00	1.00
2-MeO-ketamine	1.51	1.78	1.14
amphetamine	12.40	0.00	1.00
methamphetamine	9.06	0.00	1.00
citalopram	13.61	0.00	1.00
modafinil	2.23	2.66	1.22
praziquantel	0.47	0.56	1.10
chlorthalidone	16.37	2.43	1.25
	**MP composition**	**Flow rate (mL min^{-1})**	**Temperature (°C)**
**MP →**	**C/M/TFA/IPA 90/10/0.1/0.01**	**2.00**	**40**
**Analyte ↓**	**t_{R1} (min)**	***R***	***α***
4-F-PV8	2.87	0.00	1.00
α-PVP	4.70	0.00	1.00
4-Cl-PVP	4.33	0.00	1.00
4-F-PVP	3.45	0.00	1.00
4-MeO-*α*-PVP	6.17	0.00	1.00

4-MPrC	5.38	0.00	1.00
α-PPP	7.82	0.99	1.07
M-PPP	7.06	0.78	1.06
α-PiHP	4.04	0.00	1.00
naphyrone	6.46	0.00	1.00
TH-PVP	5.30	S	1.02
PV9	3.47	0.00	1.00
PV10	3.37	0.00	1.00
5-DBFPV	7.91	0.00	1.00
4-M-PHP	3.77	0.00	1.00
3,4-MD-PHP	4.62	0.00	1.00
4-CBC	0.97	1.04	1.08
4-CIC	5.29	0.76	1.06
4-CDC	4.59	0.00	1.00
N-ethylketamine	2.08	0.78	1.06
DXE	3.62	S	1.02
2-MeO-ketamine	2.71	2.11	1.13
amphetamine	2.31	0.00	1.00
methamphetamine	2.01	0.00	1.00
citalopram	6.05	0.00	1.00
modafinil	2.42	1.84	1.12
praziquantel	0.56	0.00	1.00
chlorthalidone	12.92	6.04	1.40
	**MP composition**	**Flow rate (mL min^{-1})**	**Temperature (°C)**
**MP →**	**C/M/DEA 90/10/0.1**	**1.00**	**40**
**Analyte ↓**	t_{R1} **(min)**	***R***	***α***
4-F-PV8	0.42	0.00	1.00
α-PVP	0.60	0.00	1.00
4-Cl-PVP	0.46	0.00	1.00
4-F-PVP	0.54	0.00	1.00
4-MeO-*α*-PVP	1.13	0.00	1.00
4-MPrC	0.98	0.00	1.00
α-PPP	1.23	0.00	1.00
M-PPP	1.41	0.00	1.00
α-PiHP	0.57	0.00	1.00
naphyrone	0.93	0.00	1.00
TH-PVP	0.96	0.00	1.00
PV9	0.54	0.00	1.00
PV10	0.54	0.00	1.00
5-DBFPV	1.63	0.00	1.00
4-M-PHP	0.65	0.00	1.00
3,4-MD-PHP	0.40	0.00	1.00
4-CBC	0.62	0.00	1.00
4-CIC	0.49	0.00	1.00
4-CDC	0.40	0.00	1.00
N-ethylketamine	0.44	0.00	1.00

DXE	1.04	0.00	1.00
2-MeO-ketamine	0.68	0.00	1.00
amphetamine	4.97	0.00	1.00
methamphetamine	3.72	0.00	1.00
citalopram	5.24	0.00	1.00
modafinil	3.89	3.67	1.29
praziquantel	0.78	0.74	1.11
chlorthalidone	O		
	**MP composition**	**Flow rate (mL min^{-1})**	**Temperature (°C)**
**MP →**	**C/M/TFA/DEA 90/10/0.2/0.02**	**1.00**	**40**
**Analyte ↓**	**t_{R1} (min)**	***R***	***α***
4-F-PV8	4.96	0.00	1.00
α-PVP	7.68	S	1.01
4-Cl-PVP	7.20	S	1.01
4-F-PVP	5.91	0.00	1.00
4-MeO-*α*-PVP	9.88	S	1.03
4-MPrC	8.95	0.50	1.03
α-PPP	12.15	1.22	1.06
M-PPP	11.20	1.04	1.06
α-PiHP	6.05	0.00	1.00
naphyrone	11.12	0.00	1.00
TH-PVP	9.02	S	1.03
PV9	6.00	0.00	1.00
PV10	5.90	0.00	1.00
5-DBFPV	13.13	S	1.02
4-M-PHP	6.50	0.00	1.00
3,4-MD-PHP	7.42	0.00	1.00
4-CBC	1.05	1.04	1.09
4-CIC	8.75	1.19	1.07
4-CDC	7.42	0.00	1.00
N-ethylketamine	3.82	1.01	1.06
DXE	6.39	S	1.02
2-MeO-ketamine	5.04	2.44	1.12
amphetamine	4.21	0.00	1.00
methamphetamine	3.38	0.00	1.00
citalopram	10.53	0.00	1.00
modafinil	5.23	2.01	1.12
praziquantel	1.28	0.72	1.08
chlorthalidone	28.71	4.64	1.32
	**MP composition**	**Flow rate (mL min^{-1})**	**Temperature (°C)**
**MP →**	**C/M/AcA/TEA 90/10/0.2/0.02**	**1.00**	**40**
**Analyte ↓**	**t_{R1} (min)**	***R***	***α***
4-F-PV8	1.26	0.00	1.00
α-PVP	2.37	0.00	1.00
4-Cl-PVP	1.44	0.00	1.00
4-F-PVP	1.96	0.00	1.00

4-MeO-*α*-PVP	5.65	0.00	1.00
4-MPrC	4.83	0.00	1.00
α-PPP	5.86	S	1.02
M-PPP	6.88	S	1.02
α-PiHP	2.17	0.00	1.00
naphyrone	4.37	0.00	1.00
TH-PVP	4.80	0.00	1.00
PV9	2.00	0.00	1.00
PV10	1.98	0.00	1.00
5-DBFPV	8.42	0.00	1.00
4-M-PHP	2.72	0.00	1.00
3,4-MD-PHP	0.94	0.00	1.00
4-CBC	1.95	0.00	1.00
4-CIC	1.41	S	1.02
4-CDC	0.94	0.00	1.00
N-ethylketamine	0.82	0.00	1.00
DXE	4.66	0.00	1.00
2-MeO-ketamine	2.71	1.87	1.15
amphetamine	17.80	0.00	1.00
methamphetamine	12.10	0.00	1.00
citalopram	19.23	0.00	1.00
modafinil	4.75	3.12	1.24
praziquantel	1.03	0.83	1.10
chlorthalidone	37.58	0.00	1.00
	**MP composition**	**Flow rate (mL min^{-1})**	**Temperature (°C)**
**MP →**	**C/M/TFA/DEA 90/10/0.5/0.01**	**1.00**	**40**
**Analyte ↓**	***t*$_{R1}$ (min)**	***R***	***α***
4-F-PV8	5.78	0.00	1.00
α-PVP	9.13	0.00	1.00
4-Cl-PVP	8.58	0.00	1.00
4-F-PVP	6.90	0.00	1.00
4-MeO-*α*-PVP	11.94	0.00	1.00
4-MPrC	10.70	S	1.03
α-PPP	14.66	1.33	1.11
M-PPP	13.54	1.00	1.10
α-PiHP	8.18	0.00	1.00
naphyrone	13.41	0.00	1.00
TH-PVP	10.88	S	1.02
PV9	7.10	0.00	1.00
PV10	7.04	0.00	1.00
5-DBFPV	16.30	0.00	1.00
4-M-PHP	7.79	0.00	1.00
3,4-MD-PHP	8.92	0.00	1.00
4-CBC	2.16	1.13	1.08
4-CIC	10.73	1.05	1.06
4-CDC	8.94	0.00	1.00

N-ethylketamine	4.55	1.09	1.07
DXE	7.69	S	1.02
2-MeO-ketamine	6.10	2.06	1.12
amphetamine	4.84	0.00	1.00
methamphetamine	3.96	0.00	1.00
citalopram	12.31	0.00	1.00
modafinil	5.27	2.02	1.11
praziquantel	1.32	0.77	1.08
chlorthalidone	X		
	**MP composition**	**Flow rate (mL min^{-1})**	**Temperature (°C)**
**MP →**	**C/M/TFA/TEA 90/10/0.5/0.01**	**1.00**	**40**
**Analyte ↓**	t_{R1} **(min)**	R	α
4-F-PV8	5.82	0.00	1.00
α-PVP	9.18	0.00	1.00
4-Cl-PVP	8.57	0.00	1.00
4-F-PVP	6.95	0.00	1.00
4-MeO-*α*-PVP	11.90	0.00	1.00
4-MPrC	10.65	S	1.02
α-PPP	14.50	1.21	1.06
M-PPP	13.36	0.97	1.06
α-PiHP	8.11	0.00	1.00
naphyrone	13.12	0.00	1.00
TH-PVP	10.66	S	1.02
PV9	7.13	0.00	1.00
PV10	6.93	0.00	1.00
5-DBFPV	15.64	S	1.01
4-M-PHP	7.66	0.00	1.00
3,4-MD-PHP	8.67	0.00	1.00
4-CBC	2.16	1.13	1.07
4-CIC	10.35	1.08	1.06
4-CDC	8.67	0.00	1.00
N-ethylketamine	4.56	1.64	1.11
DXE	7.51	S	1.02
2-MeO-ketamine	6.00	2.17	1.12
amphetamine	4.90	0.00	1.00
methamphetamine	4.22	0.00	1.00
citalopram	11.97	0.00	1.00
modafinil	5.17	1.92	1.11
praziquantel	1.29	0.65	1.07
chlorthalidone	X		
	**MP composition**	**Flow rate (mL min^{-1})**	**Temperature (°C)**
**MP →**	**C/M/TFA/DEA 90/10/0.05/0.05**	**1.00**	**40**
**Analyte ↓**	t_{R1} **(min)**	R	α
4-F-PV8	3.98	0.00	1.00
α-PVP	6.08	0.00	1.00
4-Cl-PVP	5.64	0.00	1.00

4-F-PVP	4.72	0.00	1.00
4-MeO-α-PVP	7.73	S	1.02
4-MPrC	7.02	0.52	1.03
α-PPP	9.52	0.96	1.05
M-PPP	8.55	0.79	1.04
α-PiHP	5.43	0.00	1.00
naphyrone	8.65	0.00	1.00
TH-PVP	7.03	0.50	1.03
PV9	4.71	0.00	1.00
PV10	4.67	0.00	1.00
5-DBFPV	10.04	S	1.03
4-M-PHP	5.01	0.00	1.00
3,4-MD-PHP	5.64	0.00	1.00
4-CBC	1.58	0.66	1.08
4-CIC	6.59	1.25	1.07
4-CDC	5.64	0.00	1.00
N-ethylketamine	2.98	S	1.03
DXE	4.99	S	1.07
2-MeO-ketamine	3.90	1.90	1.10
amphetamine	3.49	0.00	1.00
methamphetamine	2.84	0.00	1.00
citalopram	8.76	0.00	1.00
modafinil	4.88	1.89	1.12
praziquantel	1.16	S	1.04
chlorthalidone	26.24	3.46	1.25
**MP →**	**MP composition**<br>**C/M/A/TFA/DEA 90/8/2/0.05/0.05**	**Flow rate (mL min^{-1})**<br>**1.00**	**Temperature (°C)**<br>**40**
**Analyte ↓**	***t*$_{R1}$ (min)**	***R***	***α***
4-F-PV8	4.10	0.00	1.00
α-PVP	6.35	0.00	1.00
4-Cl-PVP	5.90	0.00	1.00
4-F-PVP	4.97	0.00	1.00
4-MeO-α-PVP	8.17	S	1.01
4-MPrC	7.38	S	1.02
α-PPP	9.90	0.78	1.04
M-PPP	9.23	0.64	1.04
α-PiHP	5.64	0.00	1.00
naphyrone	8.98	0.00	1.00
TH-PVP	7.38	S	1.02
PV9	5.01	0.00	1.00
PV10	4.91	0.00	1.00
5-DBFPV	10.61	S	1.02
4-M-PHP	5.36	0.00	1.00
3,4-MD-PHP	5.67	0.00	1.00
4-CBC	1.74	S	1.06
4-CIC	6.63	1.09	1.10

4-CDC	5.67	0.00	1.00
N-ethylketamine	3.07	0.83	1.10
DXE	5.26	0.57	1.10
2-MeO-ketamine	4.02	2.25	1.11
amphetamine	4.16	0.00	1.00
methamphetamine	3.11	0.00	1.00
citalopram	8.33	0.00	1.00
modafinil	5.69	1.77	1.11
praziquantel	1.27	S	1.07
chlorthalidone	31.38	0.00	1.00
	**MP composition**	**Flow rate (mL min^{-1})**	**Temperature (°C)**
**MP →**	**C/E/TFA/IPA 90/10/0.2/0.02**	**2.00**	**25**
**Analyte ↓**	t_{R1} **(min)**	***R***	***α***
4-F-PV8	3.10	S	1.03
α-PVP	5.34	S	1.04
4-Cl-PVP	4.90	S	1.04
4-F-PVP	3.88	S	1.02
4-MeO-*α*-PVP	7.17	S	1.06
4-MPrC	6.39	0.64	1.07
α-PPP	10.46	0.67	1.10
M-PPP	9.45	S	1.02
α-PiHP	4.68	0.00	1.00
naphyrone	7.78	S	1.04
TH-PVP	6.46	0.59	1.07
PV9	3.96	S	1.03
PV10	3.90	S	1.03
5-DBFPV	9.42	S	1.07
4-M-PHP	4.16	S	1.05
3,4-MD-PHP	6.17	0.00	1.00
4-CBC	0.87	1.17	1.17
4-CIC	6.32	1.00	1.16
4-CDC	6.13	0.00	1.00
N-ethylketamine	2.08	S	1.05
DXE	4.05	S	1.04
2-MeO-ketamine	2.72	1.72	1.20
amphetamine	2.41	0.00	1.00
methamphetamine	2.34	S	1.10
citalopram	8.97	0.00	1.00
modafinil	4.18	S	1.04
praziquantel	0.81	0.00	1.00
chlorthalidone	19.22	0.00	1.00
	**MP composition**	**Flow rate (mL min^{-1})**	**Temperature (°C)**
**MP →**	**C/E/TFA/IPA 85/15/0.1/0.1**	**2.00**	**25**
**Analyte ↓**	t_{R1} **(min)**	***R***	***α***
4-F-PV8	1.22	S	1.05
α-PVP	2.04	S	1.05

4-Cl-PVP	1.84	S	1.05
4-F-PVP	1.50	S	1.03
4-MeO-*α*-PVP	2.52	0.60	1.08
4-MPrC	2.29	0.80	1.08
α-PPP	3.64	S	1.06
M-PPP	3.28	S	1.05
α-PiHP	1.76	S	1.03
naphyrone	2.73	S	1.06
TH-PVP	2.29	0.85	1.08
PV9	1.51	S	1.04
PV10	1.48	S	1.03
5-DBFPV	3.30	0.71	1.10
4-M-PHP	1.59	0.53	1.06
3,4-MD-PHP	2.28	0.00	1.00
4-CBC	0.42	0.61	1.13
4-CIC	2.29	1.09	1.17
4-CDC	2.28	0.00	1.00
N-ethylketamine	0.87	0.00	1.00
DXE	1.45	S	1.02
2-MeO-ketamine	1.06	1.56	1.14
amphetamine	0.91	0.00	1.00
methamphetamine	0.95	0.00	1.00
citalopram	2.83	0.00	1.00
modafinil	1.84	1.39	1.18
praziquantel	0.42	S	1.11
chlorthalidone	5.26	1.61	1.36
	**MP composition**	**Flow rate (mL min^{-1})**	**Temperature (°C)**
**MP →**	**C/E/TFA/DEA 90/10/0.1/0.1**	**2.00**	**25**
**Analyte ↓**	t_{R1} **(min)**	***R***	***α***
4-F-PV8	1.17	S	1.04
α-PVP	1.88	S	1.05
4-Cl-PVP	1.72	S	1.04
4-F-PVP	1.42	S	1.03
4-MeO-*α*-PVP	2.32	0.56	1.07
4-MPrC	2.13	0.87	1.08
α-PPP	3.13	0.56	1.07
M-PPP	2.87	0.54	1.06
α-PiHP	1.63	S	1.02
naphyrone	2.54	S	1.05
TH-PVP	2.13	0.87	1.08
PV9	1.43	S	1.03
PV10	1.40	S	1.03
5-DBFPV	2.98	0.75	1.10
4-M-PHP	1.52	0.61	1.06
3,4-MD-PHP	2.03	0.00	1.00
4-CBC	0.42	1.03	1.16

4-ClC	2.03	1.30	1.18
4-CDC	2.03	0.00	1.00
N-ethylketamine	0.90	0.00	1.00
DXE	1.49	0.00	1.00
2-MeO-ketamine	1.05	1.80	1.17
amphetamine	1.02	0.00	1.00
methamphetamine	0.91	0.00	1.00
citalopram	2.49	0.00	1.00
modafinil	1.84	1.28	1.17
praziquantel	0.43	0.00	1.00
chlorthalidone	5.74	1.60	1.31
	**MP composition**	**Flow rate (mL min^{-1})**	**Temperature (°C)**
**MP →**	**C/E/TFA/TEA 90/10/0.1/0.1**	**2.00**	**25**
**Analyte ↓**	t_{R1} **(min)**	***R***	***α***
4-F-PV8	1.14	0.55	1.05
α-PVP	1.76	0.54	1.06
4-Cl-PVP	1.65	S	1.05
4-F-PVP	1.36	S	1.04
4-MeO-*α*-PVP	2.18	0.72	1.08
4-MPrC	2.01	1.06	1.09
α-PPP	2.87	0.61	1.07
M-PPP	2.67	0.57	1.07
α-PiHP	1.55	S	1.04
naphyrone	2.41	0.50	1.09
TH-PVP	2.02	1.06	1.09
PV9	1.36	0.50	1.05
PV10	1.34	0.50	1.04
5-DBFPV	2.81	1.08	1.12
4-M-PHP	1.45	0.83	1.07
3,4-MD-PHP	1.95	0.00	1.00
4-CBC	0.45	1.16	1.18
4-ClC	1.90	1.45	1.20
4-CDC	1.95	0.00	1.00
N-ethylketamine	0.88	0.56	1.06
DXE	1.52	S	1.03
2-MeO-ketamine	1.10	2.19	1.20
amphetamine	1.20	0.00	1.00
methamphetamine	1.03	0.00	1.00
citalopram	2.44	0.00	1.00
modafinil	1.89	1.22	1.19
praziquantel	0.42	0.00	1.00
chlorthalidone	6.51	1.83	1.33
	**MP composition**	**Flow rate (mL min^{-1})**	**Temperature (°C)**
**MP →**	**C/E/P/TFA/TEA 85/12/3/0.1/0.1**	**2.00**	**25**
**Analyte ↓**	t_{R1} **(min)**	***R***	***α***
4-F-PV8	1.65	0.77	1.07

α-PVP	2.53	0.67	1.07
4-Cl-PVP	2.40	0.59	1.06
4-F-PVP	1.96	0.66	1.05
4-MeO-*α*-PVP	3.20	0.93	1.09
4-MPrC	2.95	1.21	1.10
α-PPP	4.23	0.65	1.07
M-PPP	3.94	0.57	1.06
α-PiHP	2.22	0.50	1.05
naphyrone	3.55	0.64	1.07
TH-PVP	2.95	1.22	1.10
PV9	1.97	0.67	1.05
PV10	1.93	0.64	1.05
5-DBFPV	4.15	1.16	1.14
4-M-PHP	2.08	0.00	1.00
3,4-MD-PHP	2.84	0.00	1.00
4-CBC	0.62	1.27	1.19
4-CIC	2.78	1.47	1.22
4-CDC	2.84	0.00	1.00
N-ethylketamine	1.28	S	1.05
DXE	2.23	S	1.03
2-MeO-ketamine	1.58	2.35	1.22
amphetamine	1.70	0.00	1.00
methamphetamine	1.49	0.00	1.00
citalopram	3.81	0.00	1.00
modafinil	2.92	1.23	1.20
praziquantel	0.63	0.00	1.00
chlorthalidone	10.14	1.80	1.31
	**MP composition**	**Flow rate (mL min^{-1})**	**Temperature (°C)**
**MP →**	**C/E/H_2O/TFA/TEA 85/15/0.5/0.1/0.1**	**2.00**	**25**
**Analyte ↓**	t_{R1} **(min)**	***R***	***α***
4-F-PV8	0.98	0.56	1.04
α-PVP	1.57	0.58	1.04
4-Cl-PVP	1.40	0.54	1.04
4-F-PVP	1.16	S	1.03
4-MeO-*α*-PVP	1.84	1.00	1.06
4-MPrC	1.67	1.08	1.05
α-PPP	2.42	0.50	1.04
M-PPP	2.20	S	1.03
α-PiHP	1.36	S	1.01
naphyrone	2.01	0.65	1.05
TH-PVP	1.66	1.00	1.06
PV9	1.17	S	1.03
PV10	1.14	S	1.03
5-DBFPV	2.29	1.50	1.10
4-M-PHP	1.26	0.74	1.05
3,4-MD-PHP	1.52	0.00	1.00

4-CBC	0.41	S	1.05
4-CIC	1.61	1.85	1.14
4-CDC	1.53	0.00	1.00
N-ethylketamine	0.79	0.00	1.00
DXE	1.26	0.00	1.00
2-MeO-ketamine	1.01	1.53	1.10
amphetamine	0.94	0.00	1.00
methamphetamine	0.87	0.00	1.00
citalopram	1.76	0.00	1.00
modafinil	1.27	1.55	1.11
praziquantel	0.36	0.55	1.15
chlorthalidone	4.19	1.57	1.12
	**MP composition**	**Flow rate (mL min^{-1})**	**Temperature (°C)**
**MP →**	**C/E/P/H$_2$O/TFA/TEA 85/12/3/0.5/0.1/0.1**	**2.00**	**25**
**Analyte ↓**	***t*$_{R1}$ (min)**	***R***	***α***
4-F-PV8	1.04	0.70	1.04
α-PVP	1.65	0.64	1.04
4-Cl-PVP	1.48	0.63	1.04
4-F-PVP	1.23	S	1.03
4-MeO-*α*-PVP	1.97	1.07	1.07
4-MPrC	1.77	1.20	1.07
α-PPP	2.64	S	1.04
M-PPP	2.39	S	1.02
α-PiHP	1.43	S	1.03
naphyrone	2.13	0.75	1.06
TH-PVP	1.77	1.12	1.07
PV9	1.23	0.48	1.04
PV10	1.19	S	1.03
5-DBFPV	2.47	1.65	1.12
4-M-PHP	1.32	0.89	1.05
3,4-MD-PHP	1.63	0.00	1.00
4-CBC	0.41	S	1.04
4-CIC	1.71	1.86	1.14
4-CDC	1.63	0.00	1.00
N-ethylketamine	0.82	0.00	1.00
DXE	1.33	0.00	1.00
2-MeO-ketamine	1.04	1.45	1.08
amphetamine	0.98	0.00	1.00
methamphetamine	0.87	0.00	1.00
citalopram	1.98	0.00	1.00
modafinil	1.35	1.61	1.12
praziquantel	0.37	0.00	1.00
chlorthalidone	4.51	1.47	1.11
	**MP composition**	**Flow rate (mL min^{-1})**	**Temperature (°C)**
**MP →**	**C/E/P/H$_2$O/TFA/TEA 85/12/3/0.5/0.2/0.2**	**1.20**	**25**
**Analyte ↓**	***t*$_{R1}$ (min)**	***R***	***α***

4-F-PV8	1.45	0.91	1.05
α-PVP	2.28	0.85	1.05
4-Cl-PVP	2.06	0.76	1.05
4-F-PVP	1.71	0.68	1.04
4-MeO-*α*-PVP	2.66	1.40	1.12
4-MPrC	2.42	1.43	1.11
α-PPP	3.49	S	1.03
M-PPP	3.17	0.00	1.00
α-PiHP	1.99	0.50	1.05
naphyrone	2.96	1.03	1.10
TH-PVP	2.42	1.35	1.11
PV9	1.71	0.65	1.04
PV10	1.66	0.51	1.03
5-DBFPV	3.30	2.01	1.12
4-M-PHP	1.82	1.08	1.06
3,4-MD-PHP	2.20	0.00	1.00
4-CBC	0.64	0.00	1.00
4-CIC	2.30	2.15	1.15
4-CDC	2.20	0.00	1.00
N-ethylketamine	1.33	0.00	1.00
DXE	1.85	0.00	1.00
2-MeO-ketamine	1.47	1.56	1.08
amphetamine	1.38	0.00	1.00
methamphetamine	1.30	0.00	1.00
citalopram	2.79	0.00	1.00
modafinil	2.30	1.73	1.11
praziquantel	0.62	0.00	1.00
chlorthalidone	7.93	1.68	1.08
	**MP composition**	**Flow rate (mL min^{-1})**	**Temperature (°C)**
**MP →**	**C/E/P/H_2O/TFA/TEA 85/12/3/0.5/0.3/0.3**	**1.20**	**25**
**Analyte ↓**	***t_{R1}* (min)**	***R***	***α***
4-F-PV8	1.28	0.73	1.04
α-PVP	1.98	0.66	1.04
4-Cl-PVP	1.79	0.63	1.04
4-F-PVP	1.49	0.49	1.03
4-MeO-*α*-PVP	2.26	1.33	1.07
4-MPrC	2.05	1.29	1.07
α-PPP	2.97	0.00	1.00
M-PPP	2.68	0.00	1.00
α-PiHP	1.73	S	1.03
naphyrone	2.55	0.94	1.05
TH-PVP	2.05	1.21	1.06
PV9	1.49	0.51	1.03
PV10	1.44	S	1.03
5-DBFPV	2.77	1.93	1.12
4-M-PHP	1.57	0.98	1.05

3,4-MD-PHP	1.89	0.00	1.00
4-CBC	0.60	0.00	1.00
4-CIC	1.96	2.24	1.15
4-CDC	1.89	0.00	1.00
N-ethylketamine	1.16	0.00	1.00
DXE	1.62	0.00	1.00
2-MeO-ketamine	1.29	1.32	1.07
amphetamine	1.26	0.00	1.00
methamphetamine	1.19	0.00	1.00
citalopram	2.40	0.00	1.00
modafinil	2.22	1.65	1.11
praziquantel	0.58	0.00	1.00
chlorthalidone	7.69	S	1.05
	**MP composition**	**Flow rate (mL min^{-1})**	**Temperature (°C)**
**MP →**	**C/E/P/H_2O/TFA/TEA 85/12/3/0.25/0.2/0.2**	**1.20**	**25**
**Analyte ↓**	***t_{R1}* (min)**	***R***	***α***
4-F-PV8	1.54	1.05	1.11
α-PVP	2.38	0.93	1.10
4-Cl-PVP	2.20	0.84	1.10
4-F-PVP	1.81	0.81	1.05
4-MeO-*α*-PVP	2.83	1.34	1.09
4-MPrC	2.60	1.53	1.10
α-PPP	3.73	S	1.04
M-PPP	3.39	S	1.02
α-PiHP	2.08	0.61	1.04
naphyrone	3.18	1.02	1.07
TH-PVP	2.59	1.50	1.10
PV9	1.81	0.77	1.05
PV10	1.77	0.68	1.04
5-DBFPV	3.54	1.84	1.14
4-M-PHP	1.91	1.22	1.10
3,4-MD-PHP	2.41	0.00	1.00
4-CBC	0.64	0.69	1.10
4-CIC	2.41	1.97	1.11
4-CDC	2.41	0.00	1.00
N-ethylketamine	1.26	0.00	1.00
DXE	1.94	S	1.01
2-MeO-ketamine	1.47	2.03	1.13
amphetamine	1.47	0.00	1.00
methamphetamine	1.35	0.00	1.00
citalopram	3.13	0.00	1.00
modafinil	2.68	1.75	1.14
praziquantel	0.63	S	1.10
chlorthalidone	9.16	1.29	1.12
	**MP composition**	**Flow rate (mL min^{-1})**	**Temperature (°C)**
**MP →**	**C/E/P/H_2O/TFA/TEA 85/12/3/0.5/0.25/0.25**	**1.20**	**25**

Analyte ↓	t_{R1} (min)	*R*	*α*
4-F-PV8	1.36	0.81	1.04
α-PVP	2.12	0.75	1.04
4-Cl-PVP	1.92	0.70	1.04
4-F-PVP	1.51	0.59	1.04
4-MeO-*α*-PVP	2.44	1.32	1.07
4-MPrC	2.22	1.37	1.07
α-PPP	3.21	0.71	1.02
M-PPP	2.91	0.00	1.00
α-PiHP	1.85	S	1.03
naphyrone	2.73	0.98	1.05
TH-PVP	2.22	1.28	1.07
PV9	1.59	0.58	1.04
PV10	1.54	S	1.03
5-DBFPV	3.00	2.01	1.12
4-M-PHP	1.68	1.06	1.06
3,4-MD-PHP	2.03	0.00	1.00
4-CBC	0.61	0.00	1.00
4-CIC	2.11	2.12	1.15
4-CDC	2.04	0.00	1.00
N-ethylketamine	1.23	0.00	1.00
DXE	1.72	0.00	1.00
2-MeO-ketamine	1.36	1.45	1.07
amphetamine	1.31	0.00	1.00
methamphetamine	1.25	0.00	1.00
citalopram	2.56	0.00	1.00
modafinil	2.25	1.80	1.11
praziquantel	0.60	0.00	1.00
chlorthalidone	7.68	0.88	1.06
	**MP composition**	**Flow rate (mL min^{-1})**	**Temperature (°C)**
**MP →**	**C/E/P/H_2O/TFA/TEA 85/12/3/0.2/0.2/0.2**	**1.20**	**25**
**Analyte ↓**	t_{R1} **(min)**	***R***	***α***
4-F-PV8	1.57	0.94	1.06
α-PVP	2.41	0.79	1.06
4-Cl-PVP	2.24	0.77	1.05
4-F-PVP	1.86	0.75	1.05
4-MeO-*α*-PVP	2.89	1.20	1.08
4-MPrC	2.66	1.40	1.08
α-PPP	3.83	S	1.04
M-PPP	3.52	S	1.03
α-PiHP	2.11	0.52	1.04
naphyrone	3.26	0.92	1.06
TH-PVP	2.65	1.36	1.08
PV9	1.85	0.68	1.04
PV10	1.81	0.61	1.04
5-DBFPV	3.64	1.61	1.13

4-M-PHP	1.95	1.15	1.07
3,4-MD-PHP	2.48	0.00	1.00
4-CBC	0.65	0.73	1.10
4-CIC	2.49	2.03	1.20
4-CDC	2.49	0.00	1.00
N-ethylketamine	1.28	0.00	1.00
DXE	2.00	0.69	1.02
2-MeO-ketamine	1.51	2.13	1.13
amphetamine	1.53	0.00	1.00
methamphetamine	1.39	0.00	1.00
citalopram	3.36	0.00	1.00
modafinil	2.82	1.62	1.15
praziquantel	0.68	0.00	0.00
chlorthalidone	9.69	1.44	1.14
	**MP composition**	**Flow rate (mL min^{-1})**	**Temperature (°C)**
**MP →**	**C/E/P/TFA/TEA 85/12/3/0.2/0.2**	**1.20**	**25**
**Analyte ↓**	t_{R1} **(min)**	R	α
4-F-PV8	1.71	0.98	1.11
α-PVP	2.56	0.85	1.09
4-Cl-PVP	2.44	0.72	1.10
4-F-PVP	2.00	0.87	1.10
4-MeO-α-PVP	3.15	1.12	1.11
4-MPrC	2.90	1.51	1.11
α-PPP	4.18	S	1.05
M-PPP	3.87	S	1.05
α-PiHP	2.24	0.66	1.10
naphyrone	3.55	0.89	1.08
TH-PVP	2.89	1.44	1.32
PV9	2.00	0.85	1.10
PV10	1.95	0.80	1.10
5-DBFPV	4.02	1.45	1.16
4-M-PHP	2.09	1.21	1.09
3,4-MD-PHP	2.86	0.00	1.00
4-CBC	0.69	1.23	1.17
4-CIC	2.73	1.55	1.21
4-CDC	2.84	0.00	1.00
N-ethylketamine	1.31	S	1.04
DXE	2.26	S	1.02
2-MeO-ketamine	1.64	2.45	1.20
amphetamine	1.80	0.00	1.00
methamphetamine	1.57	0.00	1.00
citalopram	3.97	0.00	1.00
modafinil	3.66	1.40	1.18
praziquantel	0.72	S	1.03
chlorthalidone	12.53	S	1.04

Table S4. The effect of temperature on the enantioseparation of selected compounds on the NicoShell column. Retention time of the first eluted (t_{R1}), resolution (R), enantioselectivity (α); BP 13.79 MPa; flow rate 1.0 mL min^{-1}; UV detection 254 or 280 nm; injection volume 0.2-1.0 µL. MP composition: CO_2/MeOH/H_2O (80/20/1.0, *v*/*v*/*v*).

NicoShell			
analyte	temperature 25°C		
	t_{R1}	R	α
4-Cl-PVP	5.04	1.55	1.09
M-PPP	14.86	5.25	1.36
4-CBC	6.18	1.74	1.11
modafinil	1.12	2.56	1.25
DXE	11.30	1.30	1.08
analyte	temperature 30°C		
	t_{R1}	R	α
4-Cl-PVP	4.25	1.40	1.09
M-PPP	13.85	5.06	1.34
4-CBC	5.87	1.62	1.10
modafinil	1.10	2.50	1.23
DXE	10.44	1.24	1.08
analyte	temperature 35°C		
	t_{R1}	R	α
4-Cl-PVP	3.45	1.27	1.09
M-PPP	12.56	4.85	1.32
4-CBC	5.21	1.10	1.09
modafinil	1.09	2.42	1.22
DXE	9.34	1.16	1.08
analyte	temperature 40°C		
	t_{R1}	R	α
4-Cl-PVP	2.80	1.16	1.08
M-PPP	11.10	4.40	1.29
4-CBC	4.53	0.45	1.07
modafinil	1.08	2.39	1.20
DXE	8.23	1.08	1.07

Table S5. The effect of temperature on the enantioseparation of selected compounds on the TeicoShell column. Retention time of the first eluted (t_{R1}), resolution (R), enantioselectivity (α); BP 13.79 MPa; flow rate 1.2 mL min^{-1}; UV detection 254 or 280 nm; injection volume 0.2-1.0 µL. MP composition: CO_2/EtOH/PrOH/H_2O/TFA/TEA (85/12/3/0.25/0.2/0.2, *v*/*v*/*v*/*v*/*v*/*v*).

TeicoShell			
analyte	temperature 25°C		
	t_{R1}	R	α
4-MPrC	2.60	1.53	1.10
5-DBFPV	3.54	1.84	1.14
4-ClC	2.41	1.97	1.11

modafinil	2.68	1.75	1.14
2-MeO-ketamine	1.47	2.03	1.13
analyte		**temperature 30°C**	
	t_{R1}	R	α
4-MPrC	2.58	1.27	1.07
5-DBFPV	3.54	1.64	1.12
4-CIC	2.41	2.07	1.16
modafinil	2.68	1.83	1.14
2-MeO-ketamine	1.49	2.05	1.12
analyte		**temperature 35°C**	
	t_{R1}	R	α
4-MPrC	2.57	1.21	1.06
5-DBFPV	3.57	1.52	1.10
4-CIC	2.44	2.04	1.15
modafinil	2.70	2.02	1.14
2-MeO-ketamine	1.52	2.03	1.11
analyte		**temperature 40°C**	
	t_{R1}	R	α
4-MPrC	2.55	1.00	1.05
5-DBFPV	3.60	1.49	1.09
4-CIC	2.47	2.02	1.14
modafinil	2.74	2.03	1.13
2-MeO-ketamine	1.50	2.04	1.11

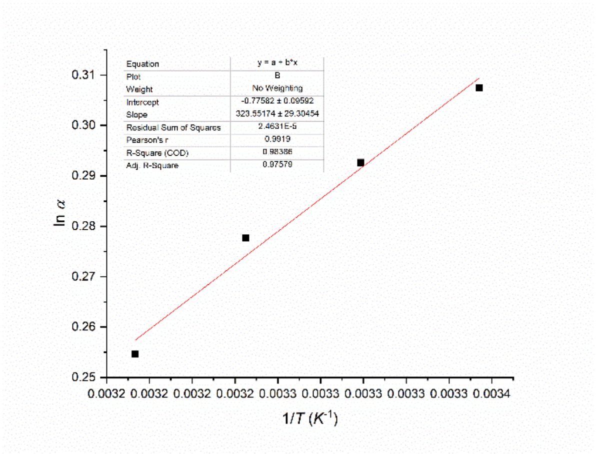

(a)

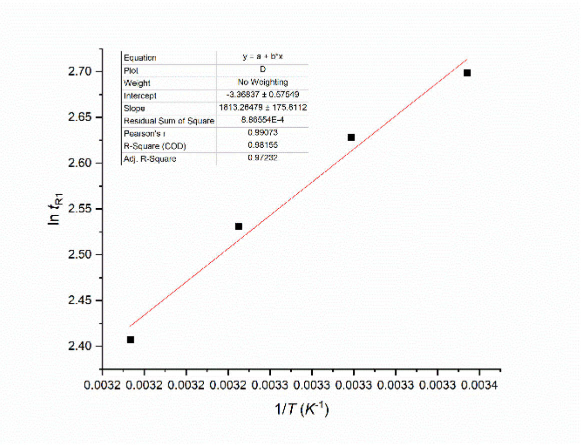

(b)

Figure S2. Dependency of ln α on $1/T$ **(a)** and dependency of ln t_{r1} on $1/T$ **(b)** of M-PPP for the NicoShell column. MP composition: CO_2/MeOH/H_2O (80/20/1.0, *v*/*v*/*v*); flow rate 1.00 mL min^{-1}; BP 13.8 MPa.

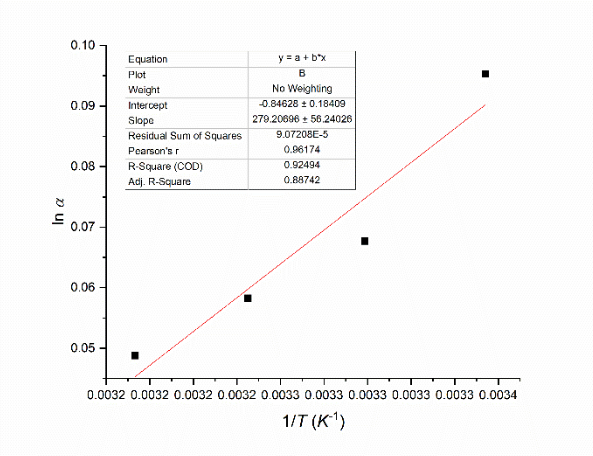

(**a**)

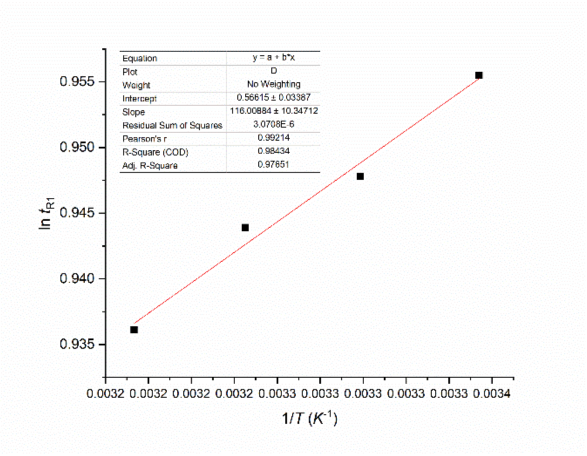

(**b**)

Figure S3. Dependency of ln α on $1/T$ **(a)** and dependency of ln t_{r1} on $1/T$ **(b)** of 4-MPrC for the TeicoShell column. MP composition: CO_2/EtOH/PrOH/H_2O/TFA/TEA (85/12/3/0.25/0.2/0.2, *v*/*v*/*v*/*v*/*v*/*v*); flow rate 1.2 mL min^{-1}; BP 13.8 MPa.
